# Spatially resolved GR-TGFβ co-activation underlies cell-type-specific signalling in triple-negative breast cancer

**DOI:** 10.1186/s12964-026-03104-7

**Published:** 2026-07-28

**Authors:** Henriett Butz, Lőrinc Pongor, Viktória Vereczki, Csaba Dávid, Kristóf Szakács, Viktória Kovács, Szonja Polett Pósa, Laura Svajda, József Tóvári, Anikó Bozsik, Vince K. Grolmusz, Tímea Pócza, János Papp, Erika Tóth, Attila Patócs

**Affiliations:** 1 Department of Molecular Genetics and National Tumorbiology Laboratory, National Institute of Oncology, Budapest, Hungary; 2 Department of Oncology Biobank, National Institute of Oncology, Budapest, Hungary; 3 HUN-REN-ONKOL-TTK-HCEMM Oncogenomics Research Group, Budapest, Hungary; 4 Department of Laboratory Medicine, Semmelweis University, Budapest, Hungary; 5 MTA–HCEMM Lendület “Momentum” Cancer Genomics and Epigenetics Research Group / Cancer Genomics and Epigenetics Core Group, HCEMM, Szeged, Hungary; 6 Department of Anatomy, Histology and Embryology, Semmelweis University, Budapest, Hungary; 7 National Academy of Scientist Education, Semmelweis University, Budapest, Hungary; 8 Department of Experimental Pharmacology and the National Tumoriology Laboratory, National Institute of Oncology, Budapest, Hungary; 9 Lendület “Momentum” Hereditary Cancers Systems Biology Research Group, Hungarian Academy of Sciences - National Institute of Oncology, Budapest, Hungary; 10 Department of Surgical and Molecular Pathology, National Institute of Oncology, Budapest, Hungary

**Keywords:** Triple-negative breast cancer, Glucocorticoid receptor signalling, Transforming growth factor beta pathway, Tumour microenvironment, Spatial transcriptomics, Cell-type-specific signalling, Tumour–immune interactions, Prognosis

## Abstract

**Background:**

The clinical relevance of glucocorticoid receptor (GR) activation in triple-negative breast cancer (TNBC) remains controversial. While preclinical studies link GR activity to tumour progression, clinical data are inconsistent, despite the routine use of synthetic glucocorticoids as supportive therapy during chemotherapy. This paradox suggests that GR signalling may be highly context- and cell-type-specific within the tumour microenvironment. Understanding the spatial organisation and molecular partners of glucocorticoid receptor signalling is therefore essential to clarify its functional and clinical impact.

**Methods:**

We comprehensively characterised spatially resolved GR expression and signalling across malignant, stromal, and immune compartments in human triple-negative breast cancer specimens. GR protein expression was assessed by immunohistochemistry, while spatial transcriptomics was used to resolve compartment-specific transcriptional programs. Stereological confocal microscopy enabled quantitative analysis of GR and transforming growth factor beta receptor 2 (TGFBR2) co-expression at single-cell resolution in a spatial distribution. These spatial data were integrated with bulk and single-cell RNA sequencing datasets to define signalling states, ligand dependence, and pathway interactions. Intercellular communication analyses were applied to identify signalling at tumour–immune interfaces. Functional relevance was assessed using in vitro migration assays and transcriptome sequencing following glucocorticoid stimulation with or without TGFBR2 inhibition. Associations with clinical outcome were evaluated using survival analyses of pathway-correlated gene signatures.

**Results:**

GR expression was enriched within the tumour microenvironment and associated with shared gene programs across tumour and stromal populations, prominently involving TGFβ pathway components. Stereological protein analyses revealed increased GR activation at invasive tumour edges and adjacent normal epithelium, accompanied by marked co-localisation with TGFBR2. Spatial transcriptomics demonstrated predominantly ligand-independent GR activation in tumour cells, whereas tumour-infiltrating immune cells displayed ligand-dependent signalling linked to local corticosteroid metabolism. Intercellular communication analyses identified enhanced GR-TGFβ signalling at the tumour–immune interface. Functionally, glucocorticoid-induced tumour cell migration was abolished by TGFBR2 inhibition. Clinically, GR–correlated and glucocorticoid-induced TGFβ gene signatures were associated with reduced overall survival.

**Conclusions:**

These findings reveal spatially regulated, cell-type-specific GR-TGFβ co-activation in TNBC. This signalling axis underlies pro-migratory tumour behaviour and adverse clinical outcome, identifying a potential prognostic marker and therapeutic vulnerability with important implications for glucocorticoid use in cancer treatment.

**Supplementary Information:**

The online version contains supplementary material available at https://doi.org/10.1186/s12964-026-03104-7.

## Abstract (plain language summary)

Triple-negative breast cancer is an aggressive form of breast cancer that lacks common hormone receptors and has limited treatment options. Glucocorticoids are steroid medicines often given to cancer patients to reduce side effects of therapy, such as nausea and inflammation. However, their effects on triple-negative breast cancer itself have been unclear and sometimes contradictory.

In this study, we examined how the glucocorticoid receptor—a protein that responds to steroid hormones—behaves in different parts of triple-negative breast tumours. Using advanced imaging and gene analysis techniques, we found that glucocorticoid receptor activity is not uniform throughout the tumour. Instead, it varies between cancer cells, immune cells, and supporting tissue, and is especially strong at the edges of tumours where cancer cells interact with their surroundings.

We also discovered that glucocorticoid receptor activity is closely linked to another signalling system called TGFβ, which is known to promote cancer cell movement and spread. In laboratory experiments, activating the glucocorticoid receptor increased cancer cell migration, but this effect disappeared when TGFβ signalling was blocked.

Importantly, tumours showing strong combined activity of the glucocorticoid receptor and TGFβ were associated with worse patient outcomes. Our findings suggest that glucocorticoid signalling can support tumour progression in a location- and cell-type-specific way.

These results help explain why glucocorticoid use in cancer care can have complex effects and highlight the need to carefully consider steroid treatment in patients with triple-negative breast cancer.

**Supplementary Information:**

The online version contains supplementary material available at https://doi.org/10.1186/s12964-026-03104-7.

## Introduction

Glucocorticoids (GCs) are widely used as adjuvant therapies in oncology to alleviate the adverse effects of chemotherapy and radiotherapy [1, 2]. Synthetic glucocorticoids, including dexamethasone, prednisolone, and betamethasone, are routinely administered to reduce nausea, oedema, allergic reactions, and treatment-related inflammation, and to improve quality of life in patients with solid tumours, including breast cancer [1, 2]. However, accumulating evidence indicates that GC signalling can also modulate tumour biology and therapeutic responses.

In breast cancer, glucocorticoid effects are strongly context-dependent and differ by hormone receptor status. In oestrogen receptor-positive (ER+) tumours, GR activation is generally associated with tumour-suppressive effects, whereas in triple-negative breast cancer (TNBC) it has been linked to tumour progression, therapy resistance, and metastasis [3, 4]. These opposing effects have been attributed, at least in part, to ER-GR heterodimerisation and the sequence similarity between oestrogen and glucocorticoid DNA-binding elements, which enables receptor cross-talk at the transcriptional level [3, 4].

Consistent with a tumour-promoting role in TNBC, high GR expression at the RNA and protein levels has been associated with poorer prognosis and reduced survival in multiple studies [5–7]. Corticosteroid use has also been linked to failure of anti-tumour therapies and adverse clinical outcomes [3, 4, 8, 9]. In contrast, other reports have failed to confirm these associations. Elkashif et al. [10] reported that GR expression predicted a favourable outcome in TNBC patients treated with taxane-free, anthracycline-based chemotherapy, while Gandhi et al. [11] and Snijesh et al. [12] showed that GR-high tumours exhibited lower proliferative indices, increased cytolytic activity, and reduced immunosuppressive T-cell populations, translating into improved disease-free and disease-specific survival. These conflicting findings highlight the complexity of GR signalling in TNBC.

One explanation for these discrepancies lies in the strong cell-type- and context-dependent nature of glucocorticoid signalling. GR responses vary across tissues and cell types, and glucocorticoids can either suppress or promote carcinogenesis depending on tumour stage, cellular context, and microenvironmental cues [3, 4, 13]. In cancer, prolonged GR activation is particularly relevant, as chronic psychological stress, pro-neoplastic inflammation, exogenous corticosteroid therapy, and intratumoral steroidogenesis can collectively suppress anti-tumour immune responses and promote tumour cell survival [3, 4, 13].

Importantly, GR activity is not exclusively ligand dependent. In TNBC, GR can be transcriptionally activated in the absence of glucocorticoids through stress-induced, p38 MAPK–dependent phosphorylation at Ser134, functioning as a cellular stress sensor [13]. This ligand-independent GR activation has been shown to regulate tumour cell migration, invasion, metabolic reprogramming, chemoresistance, and survival, thereby promoting metastatic progression [9, 13]. These findings suggest that both endogenous stress signalling and therapeutic glucocorticoid exposure converge on GR to shape aggressive TNBC phenotypes.

In this study, we aimed to define the spatial and cell type-specific landscape of glucocorticoid receptor signalling in triple-negative breast cancer. By integrating immunohistochemistry, co-localisation studies by confocal microscopy, spatial transcriptomics, single-cell and bulk transcriptomic analyses, and in vitro functional assays, we sought to characterise GR activation across malignant and stromal compartments. Our goal was to clarify how GR signalling operates within the tumour microenvironment that may contribute to disease progression.

## Methods

### Sample acquisition, tissue handling and imaging

Archived formalin-fixed paraffin-embedded (FFPE) patient samples were collected retrospectively from the Pathology Department at the National Institute of Oncology in line with the ethical permit from the Scientific and Research Committee of the Medical Research Council of the Ministry of Health in Hungary. (BMEÜ/1774-1/2022/EKU). Tissue preparation was carried out as part of routine pathological procedures. FFPE blocks that were no older than two years were selected to ensure proper RNA quality, optimised staining and pre-analytical processes for both immunohistochemistry and spatial transcriptomics. Only specimens of triple-negative histology from primary, treatment-naïve breast cancer patients were used. Pathological characterisation was conducted as part of routine diagnostics, and breast cancer subtype classification was performed as earlier reported, according to current guidelines [7, 14–16] using immunohistochemistry. Imaging was performed using the 3DHISTECH – Pannoramic 250 FLASH III Digital Scanner with 20x objective and 40x digital output (3DHISTECH, Budapest, Hungary). The images were analysed using Panoramic Digital Scanner Software 3.0.7 (3DHISTECH, Budapest, Hungary).

### Immunohistochemistry staining

Altogether 17 TNBC samples on TMA slides and individual, full TNBC slides from different patients were used for GR-total immunostaining. Immunohistochemistry was done as previously described [7]. Briefly, for total GR, a polyclonal antibody against human GR N-terminal (GTX101120, 1 mg/mL; GeneTex, Irvine, CA, USA) was applied at a dilution of 1:100 [7]. Biotinylated goat anti-mouse antibody (BA-9200 Vector Laboratories, Burlingame, CA, USA) was applied as a secondary antibody with DAB solution as the chromogen (Vector Laboratories, Burlingame, CA, USA, Impact^®^ DAB Substrate, Peroxidase (HRP). Sections were counterstained with haematoxylin (Novolink, Leica Biosystems Newcastle Ltd., Newcastle Upon Tyne, UK). Images were obtained using Nikon Eclipse 800, Olympus DP74 camera, Plan Apo S Flour 10x,20 × 0,75, Plan Flour 40x/1,30 oil, Plan Flour 63x/1,40 oil lens. For cell type identification, immunohistochemical staining was performed using the following primary antibodies: pan-cytokeratin (PanCK; clone AE1/AE3, Agilent Technologies; 1:100), CD68 (clone PG-M1, Agilent Technologies; 1:100), CD3 (clone F7.2.38, Agilent Technologies; 1:200), vimentin (clone V9, Roche Diagnostics; ready-to-use), and S100 (polyclonal rabbit antibody, Agilent Technologies; ready-to-use). All antibodies were CE-IVD certified and used according to the manufacturers’ instructions.

### Fluorescent double-staining, confocal microscopy, and spatial quantification

The same 17 TNBC on TMA slides and individual TNBC, and 6 normal breast samples, as well as 4 samples that were also used for spatial transcriptomics, were included in the fluorescent double-staining.

For double immunohistochemistry, paraffin-embedded sections were processed. Briefly, the paraffin sections were dewaxed and rehydrated with 0.05 mol/L potassium phosphate-buffered saline (KPBS). We slightly modified a previously described protocol [17]. We used the total glucocorticoid receptor (α and β) polyclonal antibody against human GR N terminal (GTX101120, GeneTex, Irvine, CS, USA) at a concentration of 1:40 and mouse TGFβR2 (TA807912, OriGene, Rockville, MD, USA) antibody against human TGFβR2 spliced transcript variant at a concentration of 1:100. The primary antibodies were diluted in KPBS + 0.4% triton-X100 and incubated for 1 h at room temperature, followed by 24 h incubation at 4 °C. After several rinses, the secondary antibodies were incubated at the same time for 3 h at room temperature. Both the Alexa Fluor488-conjugated donkey anti-mouse IgG (Cat. number: 715-545-150) and Cy3-conjugated donkey anti-rabbit IgG (Cat. number: 711-165-152) (Jackson ImmunoResearch Laboratories, Inc., USA) were diluted in KPBS + 0.4% Triton-X100 at a concentration of 1:200 in each case. After multiple washes, we cover-slipped with Vectashield DAPI (Vector Laboratories, Burlingame, CA, USA).

Multichannel confocal images were obtained using a Nikon A1R confocal microscope, 20X DIC Plan Apo VC NA = 0.75 and a 60X OIL DIC Plan Apo VC NA = 1.45 lens. Quantitative analyses were performed of the HP Z4 computer, Win10 Pro, and the Ti-S-ER motorised stage was controlled by the NIS-Elements AR 5.20.02.

### Quantitative analyses of immunohistochemistry

After we performed double immunohistochemistry of total glucocorticoid receptor and TGFβR2 counterstained with DAPI on each parallel cut slide (2095,3448, 3059,3813), we placed an about 500 μm x 500 μm virtual frame on each slide under low magnification, divided into 12 evenly sized 50–55 μm quadrangular grids. This virtual frame was on the surface area of the natural edge of the tumours, bordered by connective tissue and infiltrating immunocompetent cells. Under high magnification using the computer-controlled motorised stage, we moved the capture location along the grid, captured 12 images per slide along the superficial layer of the tumours towards the inner part of the tumours. At least 4 different captures were made at the most superficial, middle and inner layer of the tumours, altogether 500 μm deep of the tumours’ surface. Custom-written ImageJ FIJI [18] script was used to analyse the intensity of three fluorescent channels: red for total glucocorticoid receptor, green for TGFβR2 immunostaining, and blue for DAPI. First, an expert user manually placed markers on the image selecting the centres of tumour cells under the DAPI channel on each captured image. Subsequently, the script quantified the intensity of the different channels on the same image in a 10 μm circle centred on each marker. This approach allowed us to measure exclusively the intensity of the cytoplasmic and the nuclear labelling of the total glucocorticoid receptor (red channel) and the TGFβR2 (green channel) immunostaining of the previously manually identified tumour cells. For each slide, at least 500–1000 tumour cells were sampled to ensure robustness of the data [17, 19].

### Spatial transcriptomics (ST) experiment

For the spatial transcriptomics experiment, we used 4 TNBC samples, including tumour microenvironment (adipocytes, connective tissue and tumour infiltrating lymphocytes). For tissue preparation, we strictly followed the protocol of Visium CytAssist Spatial Gene Expression for FFPE – Tissue Preparation Guide (CG00518, 10x Genomics, Pleasanton, CA, USA). We applied serial sections of 5 μm using Superfrost PLUS slides (4951PLUS-001; Kalamazoo, MI, USA). RNA was extracted using Maxwell^®^ RSC RNA FFPE Kit (AS1440, Promega Corporation, Madison, WI, USA), and RNA quality was controlled using Bioanalyzer RNA 6000 Pico Kit (5067 − 1513, Agilent, Santa Clara, CA, USA). Then, slide-deparaffinization, H&E staining, imaging and decrosslinking were done exactly as described in CytAssist CG005201 protocol (CG00520, 10x Genomics, Pleasanton, CA, USA). Slide imaging was done as for immunohistochemistry (see above).

Probe hybridisation, ligation, probe release and extension were performed following the Visium CytAssist Spatial Gene Expression Reagent Kit user guide (CG000495) and with the use of the Visium CytAssist instrument and Visium CytAssist Spatial Gene Expression Kit (PN-1000441 and 1000520, respectively, 10x Genomics, Pleasanton, CA, USA). After probe-based library construction and cleaning, quality control was done by Agilent High Sensitivity DNA Kit (5067 − 4626, Agilent, Santa Clara, CA, USA) on the Bioanalyzer Instrument. Sequencing was done on Illumina Novaseq 6000 Instrument using NOVASEQ 6000 SP Rgt Kit v1.5 (100 cycles) (20028401, Illumina, San Diego, CA, USA) at a depth of ~ 250 million paired-end reads per tissue section.

### Single-cell sequencing data of triple-negative breast cancer

Single-cell RNA-seq data of TNBC samples proficient for *BRCA1* were obtained from Pal et al. [20] through the NCBI GEO database (accession numbers: GSM4909281, GSM4909282, GSM4909283 and GSM4909284), from Bassez et al. 2021 [21]. and Wu et al. 2021 [22]. Altogether, single-cell sequencing data of 37 TNBC and 13 normal breast samples (10 TNBC and 13 Normal from Pal et al. [20], 10 TNBC from Wu et al. [22], and 19 TNBC from Bassez et al. [21]. ) were reanalysed.

### Cell cultures, treatments and cell proliferation assay

All procedures were performed as previously published [23, 24]. Human triple-negative (MDA-MB231 (#92020424) and HS578T (#86082104)) were purchased from European Collection of Cell Cultures (ECACC) General Cell Collection. Cell lines were cultivated at 37 °C in a humidified atmosphere containing 5% CO2 and used until passage number 25. HS578T cells were grown in Dulbecco’s Modified Eagle’s Medium (DMEM; #10–013-CV, Corning, Corning, NY, USA) supplemented by 10% fetal bovine serum (FBS; #P40-37500 PAN-Biotech, Germany) and 1% penicillin/streptomycin (#10378016, Thermo Fisher Scientific, Waltham, MA, USA). MDA-MB231 breast mammary gland carcinoma cell lines were maintained in RPMI-1640 base medium (#BE12-702 F, Lonza Biosciences; Basel, Switzerland) using fetal bovine serum (FBS; #P40-37500 PAN-Biotech, Germany) in a final concentration of 10% and 1% penicillin/streptomycin (#10378016, Thermo Fisher Scientific, Waltham, MA, USA).

Three times a week, the cell culture medium was replaced with a fresh complete medium. When cells reached 90% confluence, they were detached from the bottom of the flask using 0.05% Trypsin-EDTA (#25300062, Invitrogen, Thermo Fisher Scientific, Waltham, MA, USA). Microscopic control and imaging were done by the EVOS M7000 imaging system using a ×10 objective.

In experimental settings, cells were kept in steroid-free media for 48 h before plating. Steroid-free media were prepared by using charcoal-stripped FBS as previously reported [13, 24]. Then, the cells were plated in six- or 24-well plates and treated with a 100nM concentration of dexamethasone (D1756, Merck, Darmstadt, Germany) and/or a 10-point dilution series of the LY2109761 TGF-β receptor type I/II (TβRI/II) dual inhibitor (SelleckChem, Houston, TX, USA) between 10⁻⁸ and 10² µM concentrations.

For determining cell viability and proliferation Sulforhodamine B (SRB) assay was used. MDA-MB-231 cells were seeded at 4,000 cells/well and HS578T at 7,000 cells/well into 96-well plates. After 24 h, cells were treated with LY210961 dissolved in serum-free medium for 72 h at 37 °C. 72 h after drug treatment, cell viability was assessed by sulforhodamine B (SRB) assay. Cells were washed twice with phosphate-buffered saline (PBS) and fixed with 10% trichloroacetic acid (TCA) for an hour at 4 °C. After fixation, cells were washed extensively with tap water and stained with 0.4% SRB solution in 1% acetic acid for 15 min at room temperature. Excess dye was removed by washing 3–4 times with 1% acetic acid, and protein-bound dye was solubilised in 200 µL of 10 mM TRIS buffer (pH 10.5). Absorbance was measured at 570 nm by Tecan Spark^®^ Multimode Microplate Reader (Tecan Group Ltd., Männedorf, Switzerland). All experiments were performed in three independent biological replicates, each with three technical replicates.

### Cell migration assay

To assess the effect of glucocorticoid receptor activation on cell migration, we used dexamethasone (DEX) as a receptor agonist, as previously described [24], and TGFβ receptor inhibitor LY2109761 at a concentration selected based on its antiproliferative effect assessment to avoid cytotoxic effect. Classic wound-healing assays were performed in 24-well plates. Twenty-four hours after treatment (DMSO, 100 nM DEX, with or without 25 µM LY2109761), the cell monolayer was scratched using a 200-µL pipette tip, and detached cells were removed by washing with phosphate-buffered saline (PBS; #21-040-CV, Corning, Corning, NY, USA). Photomicrographs were taken after 6 h, based on our previous migration dynamics optimisation experiments (Pósa et al., 2025). Images were captured using an EVOS M7000 imaging system (Thermo Fisher Scientific, Waltham, MA, USA), and wound areas were quantified with ImageJ software (https://imagej.nih.gov/ij/, Bethesda, MD, USA; last accessed 6 March 2025). Three technical replicates were included for each condition, and experiments were repeated two or three times. Wound closure (%) was calculated as: $\left(\text{wound area}_\mathrm{0h}-\text{wound area}_\mathrm{6h}\right)/\text{wound area}_\mathrm{0h}\times{100}$.

### Transcriptome output of GR-activation

We performed mRNA sequencing on two TNBC cell lines (MDA-MB213 and Hs578T) in our previous study [24] to assess the transcriptomic effects of glucocorticoid receptor (GR) activation. In the present study, we re-analysed the transcriptomic data from dexamethasone-treated MDA-MB-231 and HS578T cells [24]. Before treatment, cells were cultured in steroid-free (charcoal-stripped) medium for 24 h to eliminate endogenous hormone effects. After 24 h of dexamethasone exposure, cells were harvested, and total RNA was extracted. Library preparation and RNA sequencing were then performed. Bulk RNA-seq data analysis was conducted as previously described [24] to ensure comparability. Additionally, we performed transcriptome sequencing using Illumina Stranded Total RNA Prep with Ribo-Zero Plus library preparation kit (#20040525, Illumina, San Diego, CA, USA) following the manufacturer’s instructions on the cell lines following dexamethasone and TGFBR2 inhibitor treatment on Illumina NovaseqX instrument (Illumina, San Diego, CA, USA). We assessed previously published 24-gene pS134-GR/TGFβ1-induced signature described by Perez Kerkvliet et al. [13], which has been proposed as a surrogate for ligand-independent GR signalling. Bulk RNA-seq data analysis was conducted as previously described [24]. Single sample gene set enrichment analysis (ssGSEA) was calculated for the [13] signature using the GSVA package (v2.2.1) using the ssgseaParam (normalize set to TRUE) in R.

### Bioinformatic analysis

#### Spatial transcriptomics

Raw sequencing reads were processed using the Spaceranger tool (v2.1.1), mapping reads to the GRCh38 reference genome (refdata-gex-GRCh38-2020-A). Image alignment of spatial and microscopic images was done using Loupe Browser 7, then low-quality spots were excluded. JSON files of alignment were exported and used for the Seurat [25] package (version 5.3.1) in R (version 4.5.0) analysis.

For spot annotation, two complementary approaches were applied following the approach of Andersson et al. 2021, and Wang et al. 2024 [26, 27]: (i) transcriptomic annotation using single-cell breast tissue–specific gene expression signatures downloaded from the Human Protein Atlas (https://www.proteinatlas.org/about/download; last accessed 16 January 2024), and (ii) morphological evaluation by two observers including an experienced breast pathologist (T.E.). Cell-type enrichment per spot was first assessed by cluster heatmap analysis of the transcriptomic signatures and then cross-referenced with morphological features. Based on the integration of both approaches, each spot was finally manually annotated as tumour cells, fibrocytes/fibroblasts, adipocytes, tumour-infiltrating lymphocytes, macrophages, or mixed populations.

#### Single-cell sequencing

Single-cell RNA-seq data of TNBC samples proficient for *BRCA1* were obtained from Pal et al. [20] (GSE161529), and Wu et al. 2021 [22] (GSE176078) through the NCBI GEO database, while the data of Bassez et al. 2021 [21] was obtained from https://lambrechtslab.sites.vib.be/en/single-cell. Altogether, single-cell sequencing data of 37 TNBC patients were reanalysed. For normal breast tissue, cell-type-specific sc-seq data of 47,662 cells were downloaded from ProteinAtlas (proteinatlas.org/humanproteom/tissue+cell+type/breast, last accessed 30 December 2022).

Samples were imported with Seurat (v5.3.1) [25], filtering each sample with subset comment with the following options: nFeature_RNA > 200 & nFeature_RNA < 2500 & percent.mt < 5. Samples were then merged, normalized and scaled with NormalizeData, FindVariableFeatures and ScaleData functions. Clusters were identified with the RunPCA, FindNeighbors, RunTSNE, RunUMAP and FindClusters with default settings. Sample layers were joined using the JoinLayers function. Data was exported to h5ad format using the reticulate and SeuratDisk packages. Cells were annotated with CellTypist (v1.7.1) [28] using Immune_high, Immune_low and Cells_Adult_Breast annotations. In case of immune cells annotated by the broaded Immune_high category, we assigned to each Seurat cluster the cell type based on the majority of immune cell annotations in each cluster. Similarly, for epithelial cells, we assigned to each seurat cluster the Cells_Adult_Breast category with majority cell assignments. In case of luminal type cells, we replaced them with “TNBC” in case of tumor samples and to “Epithelial” in case of normal samples. Furthermore, we annotated a cell cluster as adipocyte based on marker expression (such as FABP4). Gene set enrichment for GR and TGFβ gene sets was calculated using the escape [29] package (version 2.4.0), using the „UCell” method with default settings.

#### Cell-cell communication analysis

We analysed cell-cell ligand-receptor interactions using CellChat v2.2.0 [30]. We created a spatial CellChat object with the normalised Spatial expression data slot, metadata and coordinates from all patients, grouping the tumour spots by TCs and the TME spots by cell type. Then, we performed a cell–cell communication analysis using the full database available with CellChat (CellChatDB.human) using default settings for all steps on the merged sample dataset. Cell-cell interaction data were then subjected to Cytoscape (v3.2.1), where the cell communication network was analysed, and results were visualised.

#### Prediction of clinical outcome based on GR and TGFβ activation

To predict clinical outcome, we used individual GR (*NR3C1* gene) and *TGFBR2* gene, GR-activated and TGFβ signature, and cell-specific (breast cancer cell, breast cancer adipocyte, breast cancer fibroblast, breast cancer infiltrating lymphocytes and macrophages) gene sets correlating with the expression of *NR3C1* identified by our ST experiment. We then calculated gene set activities using the ssGSEA functions of SurvSig [31] using the TCGA BRCA dataset, subsetting to the TNBC (Basal) tumours (*n* = 189). ssGSEA gene set activities were calculated using the “ssGSEA compare”, while ssGSEA-based survival was computed using the “ssGSEA Survival” tab. Single-gene-based survival was calculated using the “Gene Survival” tab. In all survival analyses, the TNBC samples were selected from the TCGA BRCA cohort.

#### Gene set enrichment analysis

For pathways and gene ontology categories was done using Metascape and ToppGene Suite algorithms [32, 33]. Statistically enriched terms (can be GO/KEGG terms and canonical pathways) were identified, and accumulative hypergeometric p-values and enrichment factors were calculated and used for filtering. The remaining significant terms were then hierarchically clustered into a tree based on Kappa-statistical similarities among their gene memberships. Then 0.3 kappa score was applied as the threshold to cast the tree into term clusters. The term with the best p-value within each cluster was selected as its representative term, and it was displayed in a dendrogram. The heatmap cells are coloured by the p-values; white cells indicate the lack of enrichment for that term in the corresponding gene list.

#### *Gene overlap analysis*

Overlaps were illustrated on circos plots. On the outside, each arc represents the identity of each gene list. On the inside, each arc represents a gene list, where each gene member of that list is assigned a spot on the arc. Purple lines link the same genes that are shared by multiple gene lists (notice a gene that appears in two gene lists will be mapped once onto each gene list; therefore, the two positions are purple-linked). The greater the number of purple links, implies greater the overlap among the input gene lists [33].

### Data availability

All data are presented in the manuscript; public databases from which validation data were obtained are indicated in the Methods section. Transcriptomic data will be deposited in publicly available database upon acceptance.

## Results

### GR expression is enriched in the tumour microenvironment of breast cancer

**Fig. 1:**
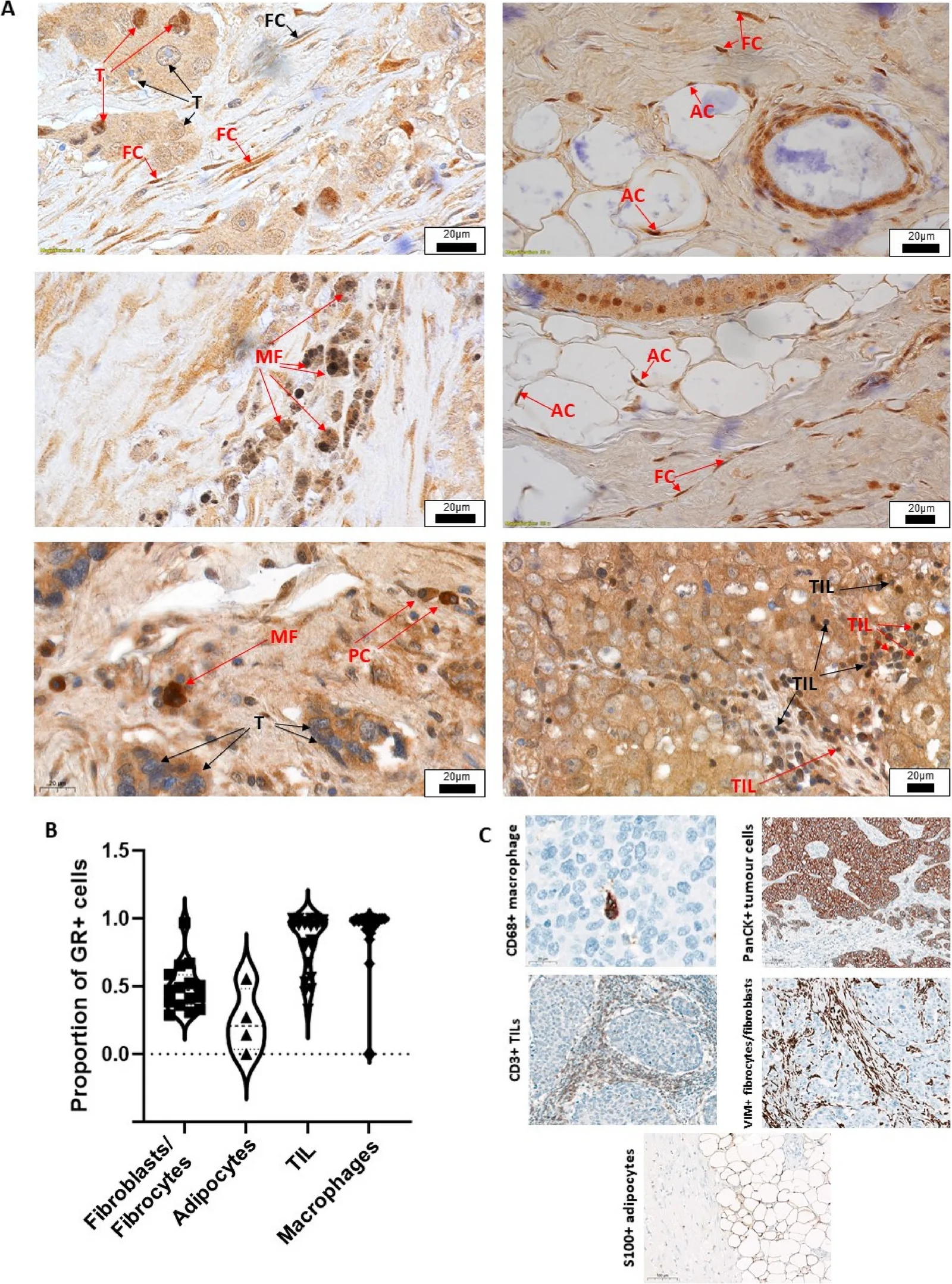
GR immunostaining in different breast cancer cell types. **A** GR immunostaining using an antibody against total GR was strong in connective tissue within and adjacent to triple-negative breast cancer cells. Tumour cells (T) showed moderate cytoplasmic and variable nuclear staining. Among TME cells, fibrocytes/fibroblasts (FC), adipocytes (AC), lymphocytes (TIL), and macrophages (MF) showed strong positivity. Red indicated GR positive staining, black indicates GR-negative staining (**B**) TMA cell quantification indicated heterogeneity in the proportion of GR-positive cells relative to total cell number. (**C**) Used cell markers for accurate cell type identification

GR expression in tumour epithelial cells of 161 triple-negative breast cancer (TNBC) samples showed strong association with patient survival [7]. In addition, prominent GR expression was observed in the tumour microenvironment of a subset of tumours, too. Therefore, GR expression was assessed in 17 samples, a total of 1,391 fibroblasts/fibrocytes, 30 adipocytes, 1,304 tumour-infiltrating lymphocytes (TILs), and 996 macrophages were quantified. Using an antibody detecting total GR, we observed strong but heterogeneous GR positivity across these tumour microenvironment components defined by using cell-type markers as well (Fig. 1A-C).

**Fig. 2:**
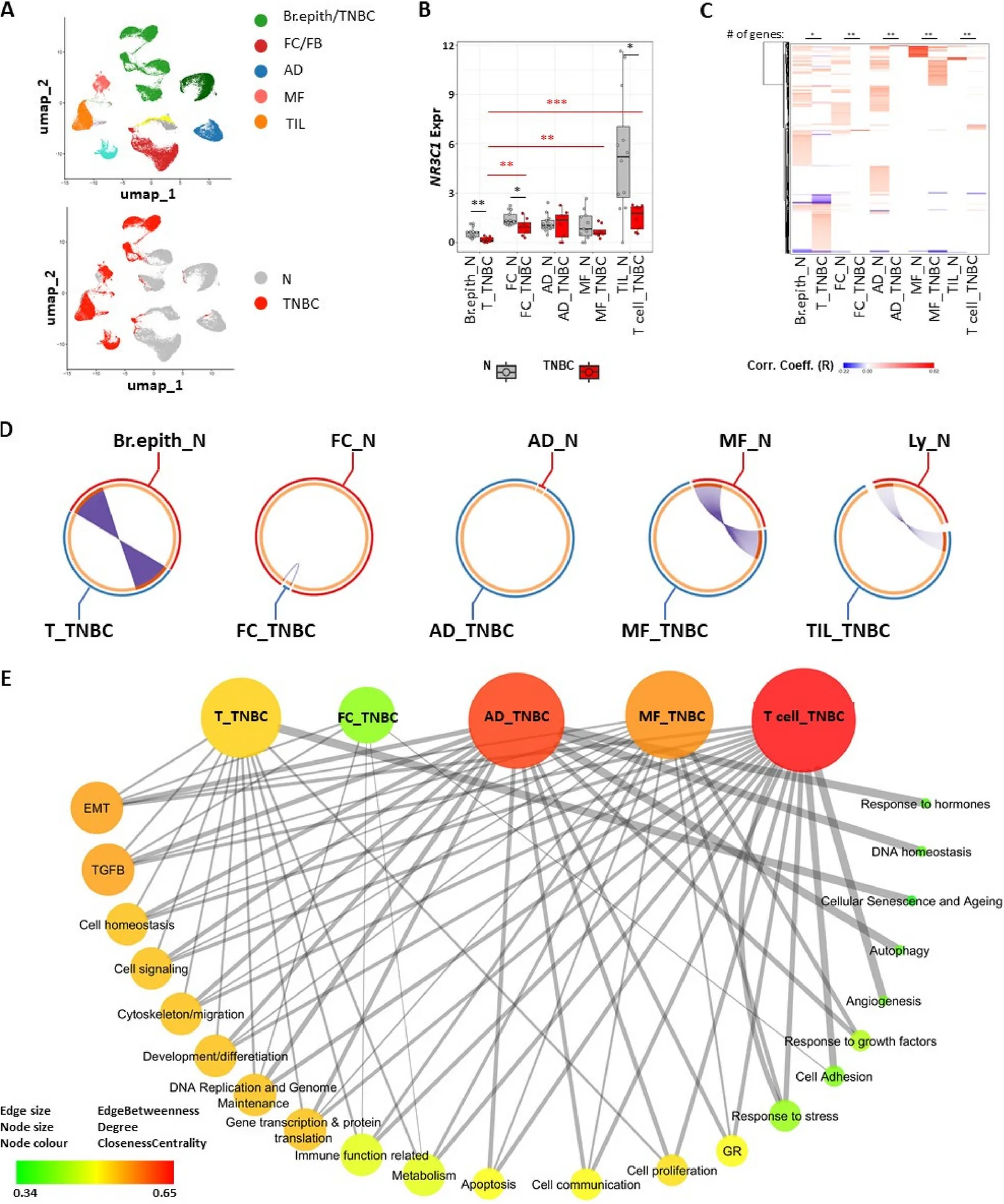
GR expression and biological function in different breast cancer cell types. **A–B** Single-cell RNA sequencing data reveal a stronger expression of the GR-encoding gene *NR3C1* in tumour microenvironment (TME) cells compared to triple-negative breast cancer (TNBC) cells. Statistical comparisons were done by Wilcoxon test, *: *p* < 0.05; **: *p* < 0.01; ***: *p* < 0.001. **C** GR-correlated genes display distinct expression patterns and numbers of differentially expressed genes between tumour and normal tissue across different cell types. Color scale indicates Spearman R correlation coefficient. Statistical comparisons were done by Wilcoxon test, *: *p* < 0.05; **: *p* < 0.01. **D** Gene overlap analysis shows GR signalling functions were most preserved in adipocytes and least preserved in tumour cells. The Circos plot illustrates shared genes (indicated by purple lines) across tumour vs. normal tissue comparisons. On the outside, each arc represents the identity of each gene list (red: normal; blue: tumour). On the inside, each arc represents a gene list, where each gene member of that list is assigned a spot on the arc. Dark orange colour represents the genes that are shared by multiple lists and light orange colour represents genes that are unique to that gene list. Purple lines link the same gene that are shared by multiple gene lists. The greater the number of purple links and the longer the dark orange arcs implies greater overlap among the input gene lists. **E** Functional enrichment analysis of GR-correlated genes differentially expressed between tumour and normal tissue. Node size in the network reflects the degree of connectivity among biological functions and pathways across cell types

To validate these semi-quantitative IHC findings, we re-analysed single-cell RNA-sequencing data from breast cancer (published by Pal et al.) including*13 normal* and *10 TNBC* specimens [20]. Consistent with the protein-level results, *NR3C1* (encoding GR) expression was enriched in TME cell types (Fig. 2A–B). Because GR functions as a nuclear receptor (regulating expression of genes containing glucocorticoid-responsive elements in their promoter regions) genes whose expression correlated with *NR3C1* could be considered putative GR-regulated targets [12, 34–36]. When comparing tumour vs. normal tissues across cell types, *NR3C1*-correlated genes differed in both number and expression patterns (Fig. 2C; Supplementary Figure SF1A-C). Gene-overlap analysis further supported these findings (Fig. 2D). Specifically, the extent of overlap between GR-regulated gene sets in tumour and matched normal cell populations is reflected by the number of shared genes (purple links) and shared gene segments (dark orange arcs). These analyses demonstrate that GR-regulated transcriptional programs were slightly preserved in immune and stromal cell populations, and tumour epithelial cells exhibited reduced overlap, indicating extensive rewiring of GR signalling during malignant transformation. After identifying cell-type-specific differences in GR-correlated genes between tumour and normal tissues (Wilcoxon test, FDR-adjusted *p* < 0.05), we focused on TNBC-specific correlations. Enrichment analysis of tumour-specific GR-correlated gene sets across different cell types revealed shared biological processes and pathways, including TGFβ signalling, epithelial-mesenchymal transition (EMT), cellular stress responses, and immune-related signalling (Fig. 2E).

### Spatial distribution and co-localisation of GR and TGFBR2 proteins reveal increasing GR activation towards TNBC tumour edges and adjacent normal breast epithelium

**Fig. 3:**
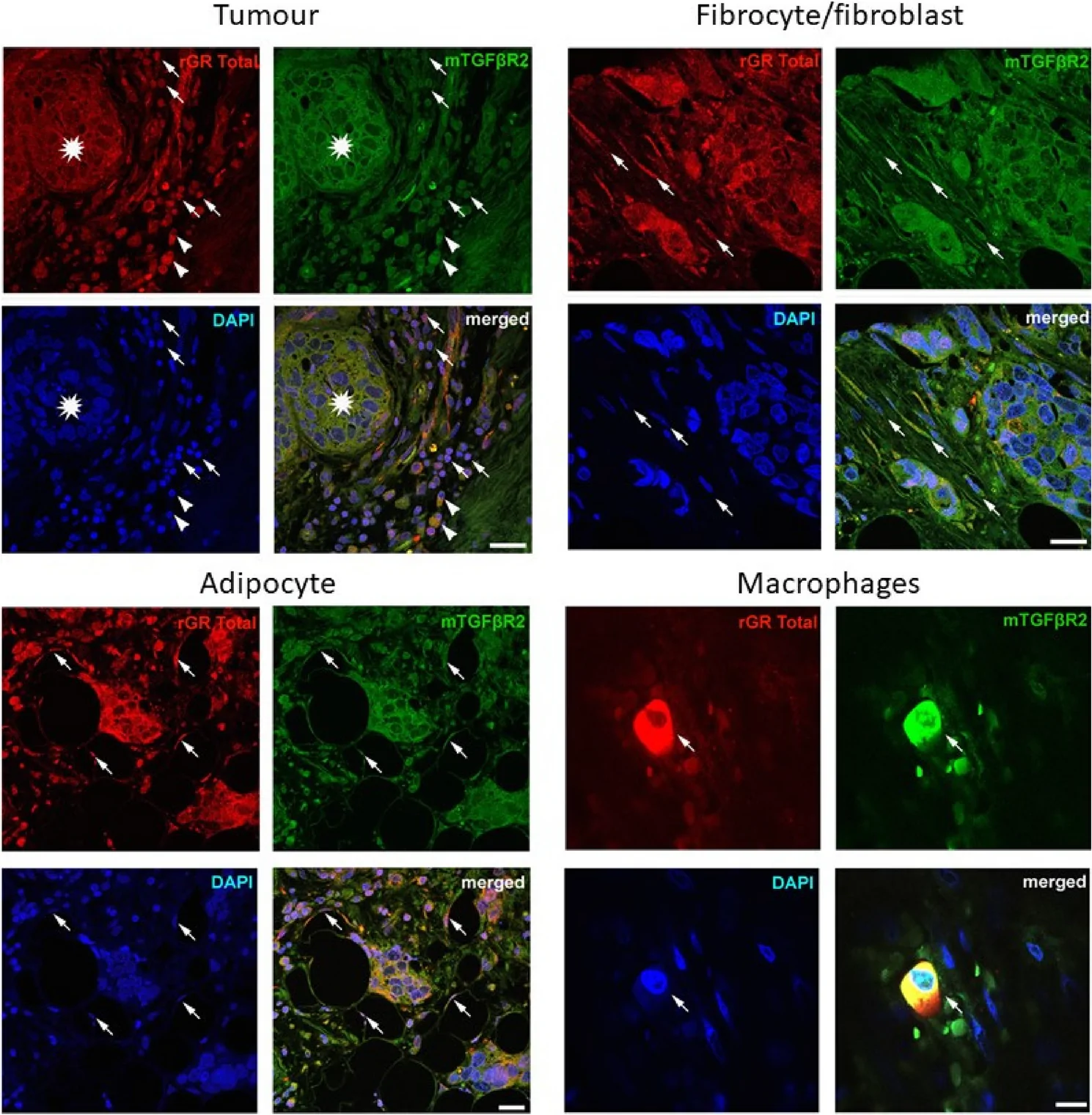
Co-localisation of GR and TGFBR2 in TNBC cells and the tumour microenvironment (TME) visualised by confocal microscopy. Red fluorescence: GRt (GR-total); green: TGFBR2; blue: DAPI-stained nuclei GR-TGFBR2 co-localisation was observed in both tumour (white star) and TME cells (white arrows), with macrophages showing robust staining. T: tumour (TNBC); CT: connective tissue with fibroblasts; M: macrophages Scale: 20 μm

Because TGFβ signalling emerged as one of the main pathways associated with GR-correlated genes on the RNA level, we next examined GR and TGFBR2 protein expression. We investigated whether these proteins are co-localised within the same cells using double immunostaining and confocal microscopy on four TNBC samples (later used for spatial transcriptomics) and on six additional breast tissue specimens, including three normal specimens from non-tumour breast samples, two normal samples adjacent (< 2 cm) to TNBC lesions, and one normal sample located more than 2 cm from a TNBC lesion. GR-TGFBR2 co-localisation was observed in both tumour and TME cells, with particularly strong co-expression in macrophages (Fig. 3).

**Fig. 4:**
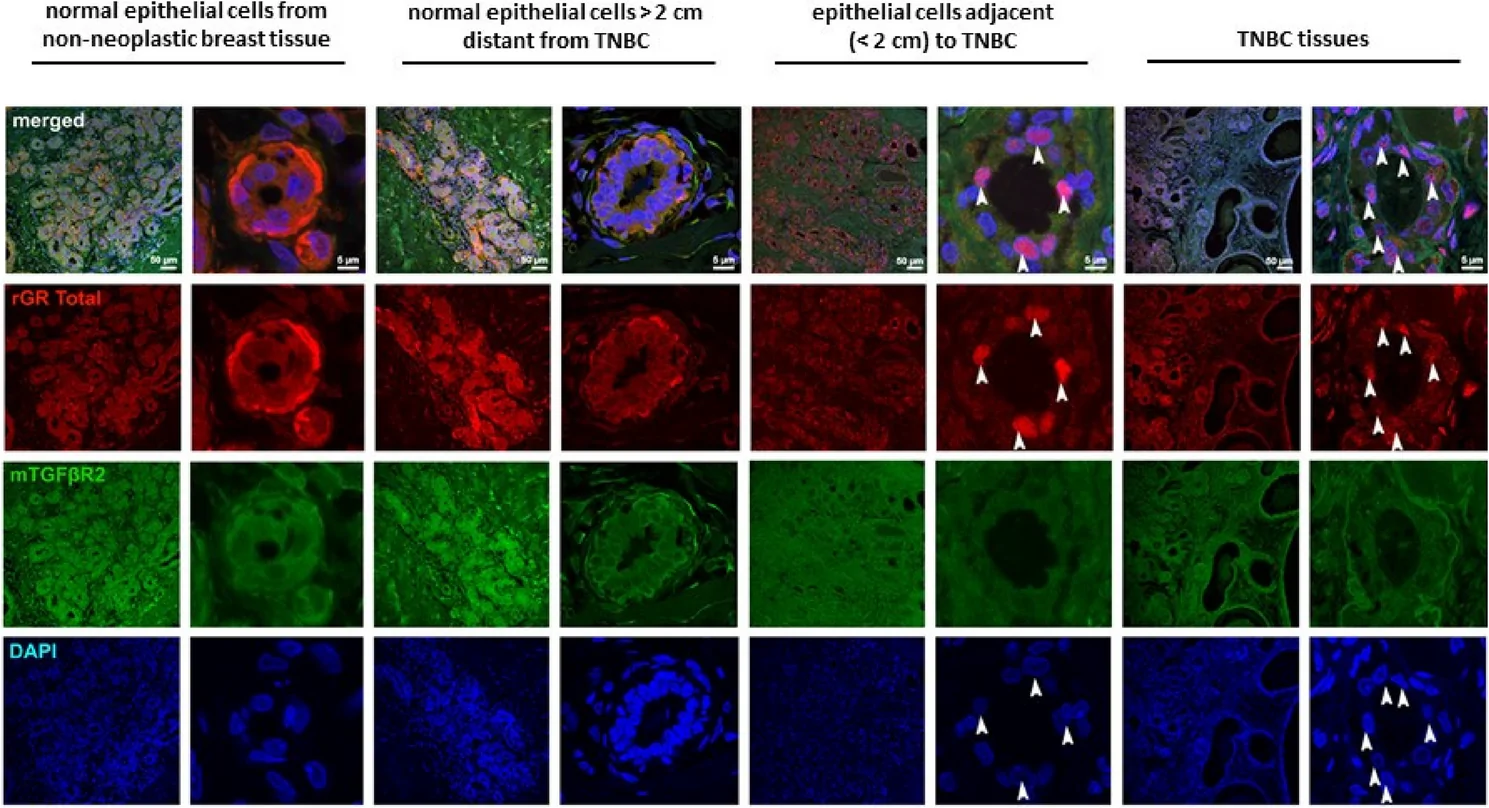
Nuclear localisation of GR in breast specimens visualised by confocal microscopy. Red fluorescence: GRt; green fluorescence: TGFBR2; blue: DAPI-stained nuclei GR staining in: (i) normal epithelial cells from non-neoplastic breast tissue (*n* = 3), (ii) normal epithelial cells > 2 cm distant from TNBC (*n* = 3), (iii) epithelial cells adjacent (< 2 cm) to TNBC (*n* = 3), and (iv) TNBC tissues (*n* = 3). In normal breast epithelial cells, strong cytoplasmic. GR staining was observed. Nuclear localisation of GR was detected in epithelial cells adjacent to TNBC and within TNBC tissues. White arrowheads indicate nuclear GR staining

Confocal microscopy revealed predominantly cytoplasmic GR localisation in normal breast epithelial cells. In contrast, nuclear GR staining – indicative of receptor activation – was detected in epithelial cells adjacent to TNBC and within tumour epithelial cells, suggesting GR activation in both the tumour and the surrounding tissue (Fig. 4).

**Fig. 5:**
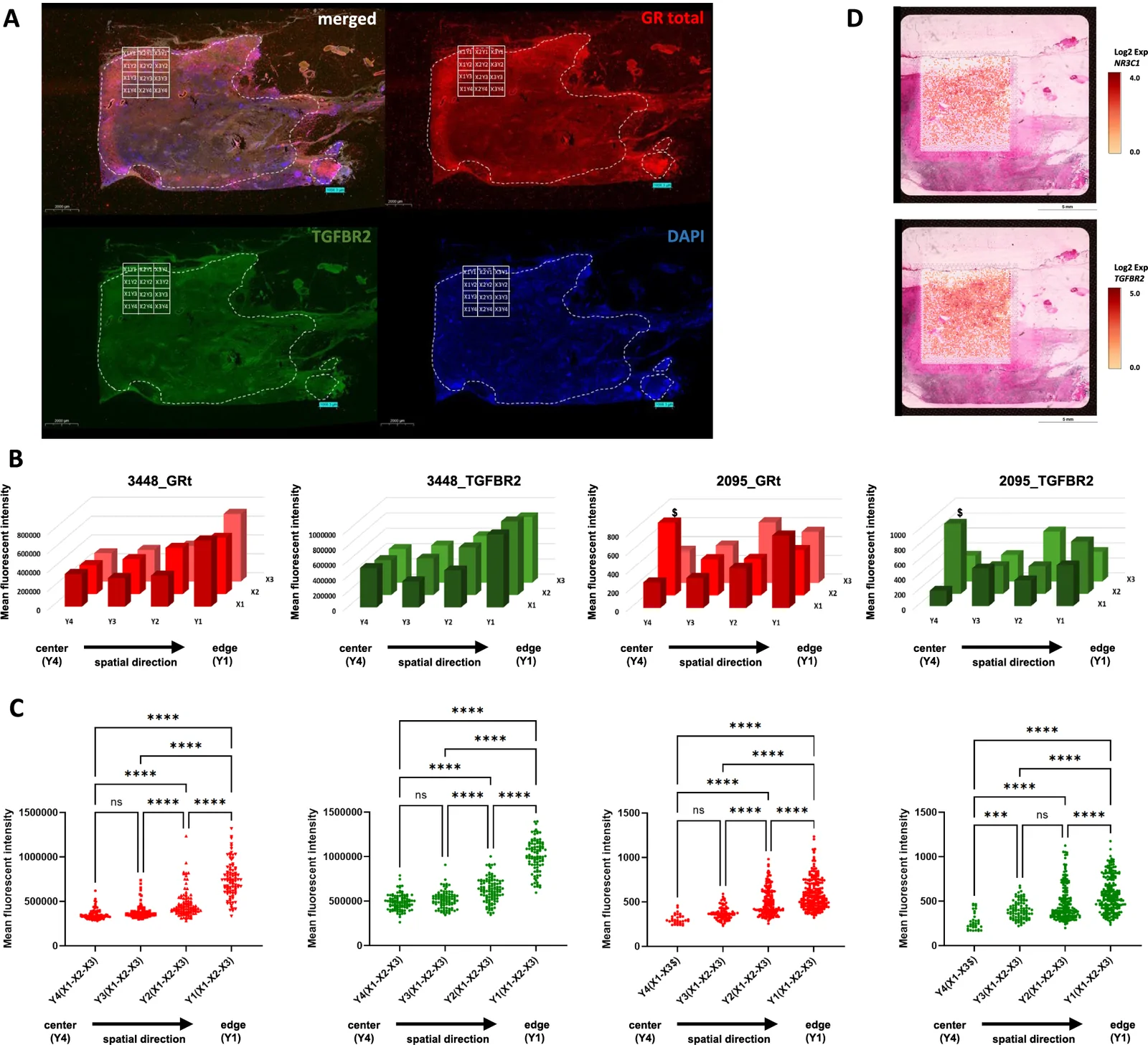
Stereology based analysis of GR and TGFBR2 in TNBC tissues. **A** Confocal microscopy of double staining for GR-total (GRt) and TGFBR2. Regions of interest (ROIs) correspond to both spatial transcriptomic and stereology based protein quantification images. Red fluorescence: GRt; green fluorescence: TGFBR2; blue: DAPI-stained nuclei. **B** Three-dimensional quantification of GRt and TGFBR2 staining in two independent TNBC specimens (#3448 and X2095). $ indicates Y4 × 1 localisation, where signal intensity was outlier. **C** Quantification of GRt and TGFBR2 staining from tumour centre to edges (direction “X” merged as Y4 × 1–4 → Y1 × 1–4). Statistical analysis was performed using Kruskal–Wallis ANOVA with Dunn’s post-hoc test. $: Y4 × 2 was excluded from statistical analysis as its signal was severely outlier due to an unknown reason. ***: *p* ≤ 0.001; ****: *p* ≤ 0.0001. **D** Spatial transcriptomic images also showing *NR3C1* (encoding GR) and *TGFBR2* expression, with stronger signals observed from the tumour centre toward the edges

To assess the protein expression in a spatial context, we performed quantitative stereological analysis of GR and TGFBR2 using dual-fluorescence confocal microscopy. Quantification along the tumour centre-edge axis was feasible in two specimens owing to their structural integrity and availability for spatial transcriptomics. In both cases, protein and transcriptomic data consistently demonstrated increased expression of GR and TGFBR2 from the tumour centre toward the edge (Fig. 5A–C). Spatial transcriptomics data further confirmed a gradual increase in *NR3C1* expression from the tumour centre to the edge (including the two samples: 3448 and 2095 used for stereological analysis of protein staining) (Fig. 5D).

### Spatial distribution of GR activation in breast cancer highlights interaction with TGFβ signalling

To explore the spatial architecture of GR activation, its relationship to *TGFBR2*, and its potential role in cell–cell interactions, we performed spatial transcriptomic analysis on four TNBC samples. Following the approach of Andersson et al. 2021, and Wang et al. 2024 [26, 27], each spot was annotated by integrating morphological evaluation with transcriptomic signatures, allowing classification into tumour, TME-fibroblasts/fibrocytes, TME-adipocytes, and tumour-infiltrating immune cells (TIICs) (see details in the methods section). Because each spot represented an average of 15–30 cells, mixed categories were also assigned.

To estimate GR activity in the different compartments, we performed correlation analyses between *NR3C1* and other genes within each annotated category, followed by Gene Set Enrichment Analysis (GSEA) of Gene Ontology (GO) biological process terms. Only genes common to at least three of the four samples were included (Supplementary Table ST1). GR-correlated genes displayed distinct expression patterns among cell types/compartments (Supplementary Figure SF2A). In tumour cells, GR primarily regulated processes related to cell proliferation, adhesion, migration, cellular homeostasis, metabolism, and cell communication. In adipocytes, GR activity was linked to lipid biosynthesis and metabolism, development and differentiation, and mesenchymal stem cell migration. In tumour-adjacent fibroblasts, GR was associated with cellular stress responses and apoptosis, whereas in TIICs, GR influenced cytoskeleton organisation and several immune-related functions (Supplementary Figure SF2A).

Focusing on tumour cells, first we compared tumour vs. normal breast epithelial spots. Beyond the expected processes, both glucocorticoid and TGFβ signalling pathways were significantly enriched (Supplementary Table ST2A, Supplementary Figure SF2B). Analysis of GR-correlated genes identified 606 genes unique to normal epithelium, 3,983 genes unique to tumour cells, and 1,740 shared genes. In normal breast tissue, GR-regulated epithelial morphogenesis, development, and proliferation, in association with oestrogen receptor (ESR1) signalling. In contrast, in tumour cells, GR predominantly influenced cell proliferation, migration, immune-related pathways, and TGFβ signalling (Supplementary Table ST2B), indicating a functional shift in GR activity during tumour progression.

**Fig. 6:**
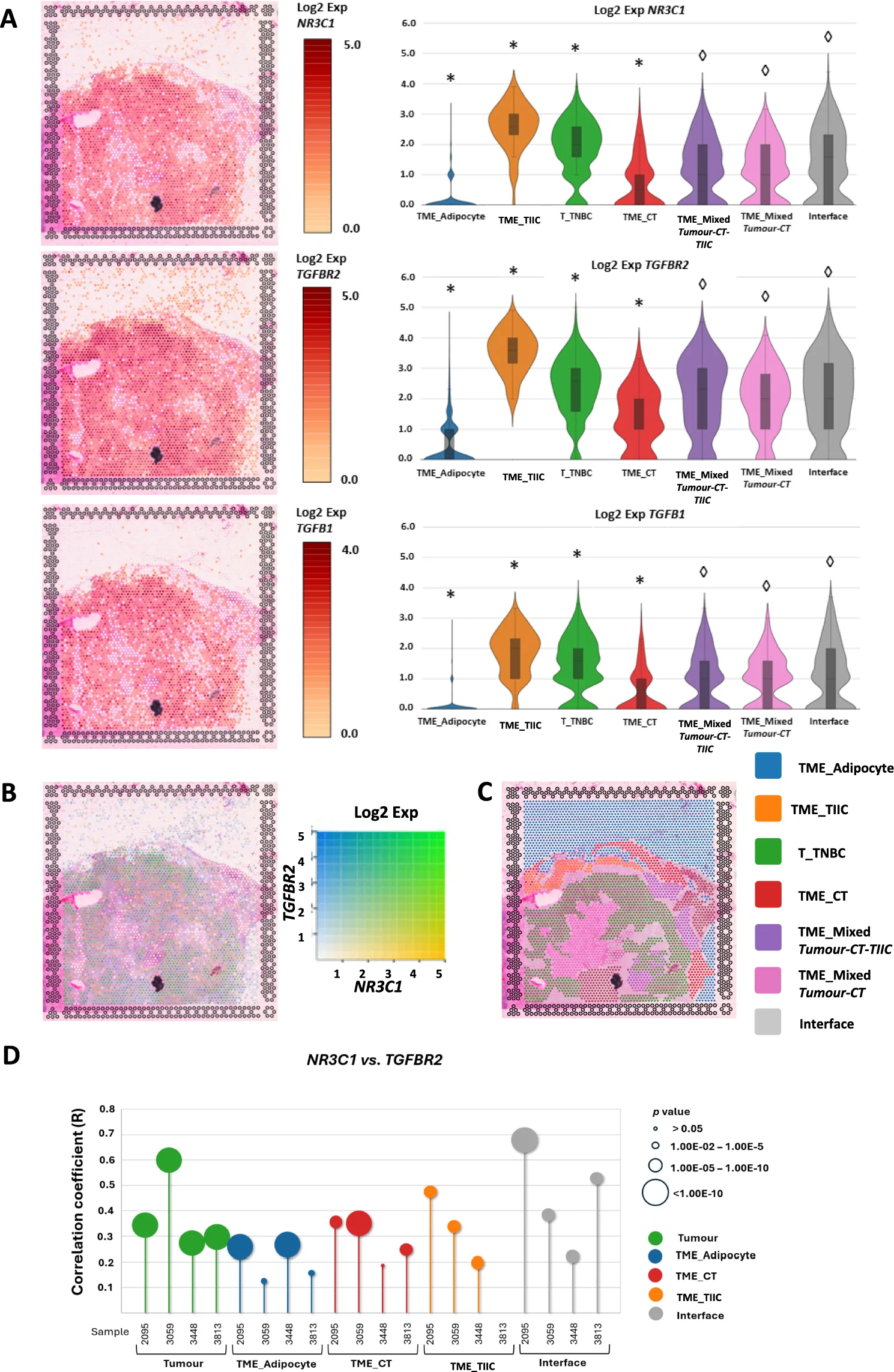
Spatial correlation between *NR3C1*,* TGFBR2*, and *TGFB1* expression in TNBC. **A–C** Representative spatial transcriptomic images showing significant correlations between *NR3C1* and *TGFBR2/TGFB1* expression across distinct tissue compartments (“spot types”) An asterisk (*) indicates a significant difference (*p* < 0.05) compared with all other spot types; a diamond (◊) denotes a significant difference (*p* < 0.05) compared with “pure” spots (tumour, adipocyte, connective tissue, or TIL). Statistical analysis was performed using Kruskal–Wallis ANOVA with Dunn’s post-hoc test and Bonferroni correction for multiple comparisons. **D** Spearman correlation coefficients of *NR3C1* with *TGFBR2* and *TGFB1* across all investigated samples. Lollipop head size represents *p*-values, and colours indicate different tissue compartments

When focusing exclusively on tumour regions, analysis of GR-correlated genes common to all four samples (11,515 genes) again revealed TGFβ signalling as a significantly enriched pathway, consistent with single-cell sequencing results (Supplementary. Table ST2C). Accordingly, we next examined TGFβ signalling in spatial context, and found that in all four specimens, *TGFBR2* and *TGFB1* expression showed significant correlation with *NR3C1* across different cell types (Fig. 6A-D).

### Local cortisol activation and tumour–stroma signalling interactions involving GR and TGFβ

Glucocorticoids circulate in the blood bound to corticosteroid-binding globulin (CBG) and must be released to exert biological effects in peripheral tissues [37]. Neutrophil elastase (NE, encoded by *ELANE*) – secreted predominantly by neutrophils and other immune cells – is the major enzyme known to cleave CBG [37]. Chymotrypsin has also been reported to release cortisol through CBG cleavage [38], although we observed no expression of its encoding gene in normal breast or TNBC samples. Once released, corticosteroids diffuse intracellularly, where 11β-hydroxysteroid dehydrogenase type 1 (*HSD11B1*) converts inactive cortisone into active cortisol, whereas *HSD11B2* catalyses the reverse reaction [3].

**Fig. 7:**
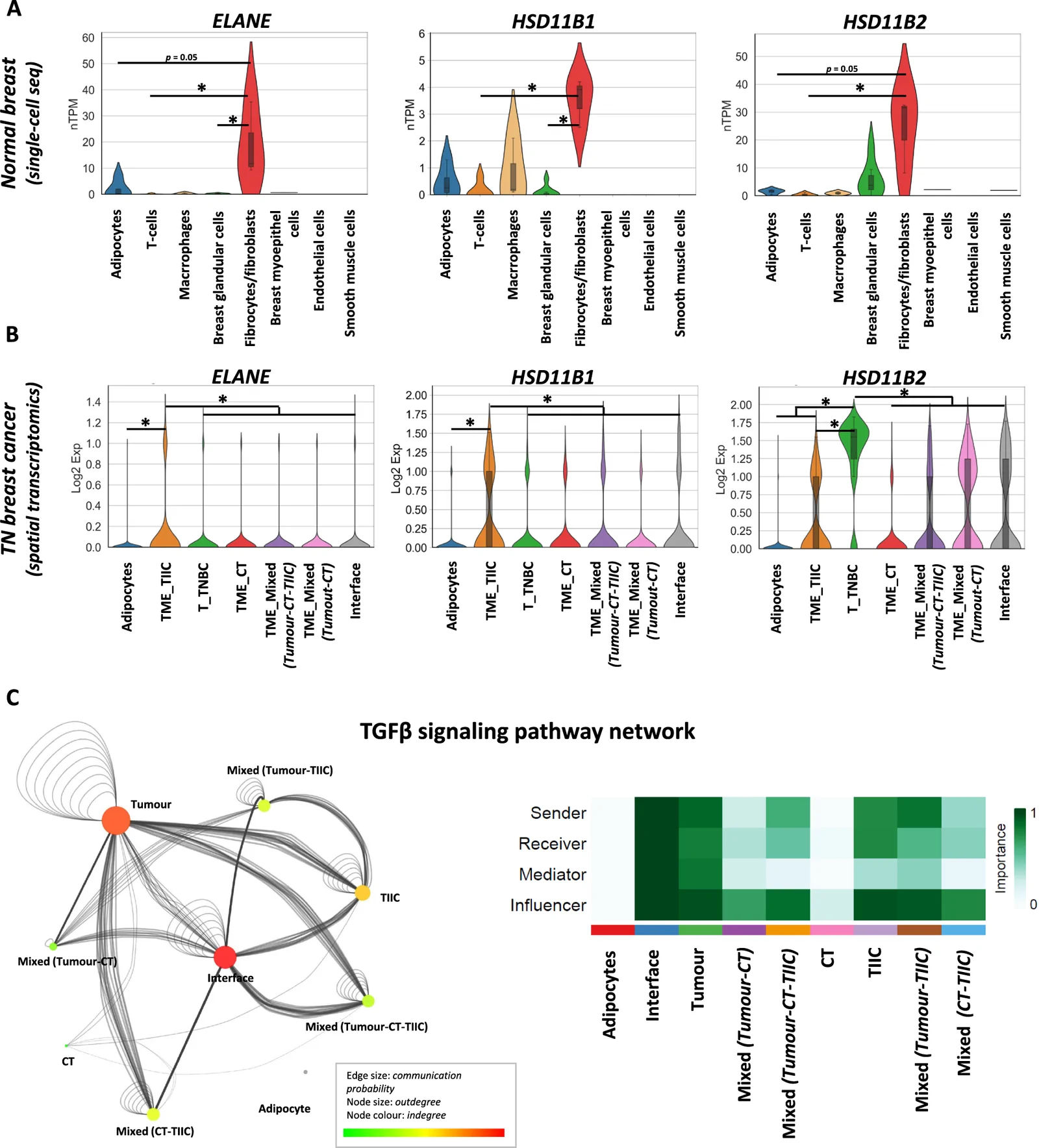
Cell–cell communication between TNBC tumour and tumour microenvironment (TME) components. **A–D** In normal breast tissue, *HSD11B1* expression is significantly elevated in fibrocytes, whereas in TNBC it is predominantly expressed in tumour-infiltrating immune compartment (TIICs). Comparisons were done using Kruskall-Wallis analysis of variance with Dunn’s posthoc test, where * indicated *p* < 0.05. **E** TGFβ signalling plays a major role in cell-cell communication between TNBC and TILs, as indicated by integrated CellChat analysis visualized in Cytoscape. In contrast, communication among connective tissue, adipocytes, and mixed compartments lacking TNBC or TIL components shows negligible involvement of TGFβ signalling

To assess local steroid bioavailability within breast cancer tissue, we analysed our spatial transcriptomic data and complemented this with single-cell sequencing data of normal breast tissue from the Human Protein Atlas. In the normal breast, *ELANE* expression was highest in fibrocytes, indicating that cortisol activation occurs primarily in the stromal compartment (Fig. 7A, Supplementary Figure SF3A). In TNBC, by contrast, *ELANE* was most strongly expressed in tumour-infiltrating immune cells (TIICs) (Fig. 7B, Supplementary Figure SF3B).

*HSD11B1* was primarily expressed in connective tissue fibrocytes and fibroblasts in normal breast, consistent with stromal cortisol activation (Fig. 7A). In TNBC, however, tumour cells showed high *HSD11B2* and low *HSD11B1* expression, indicating ligand-independent GR activation. Within the tumour microenvironment, *HSD11B1* was elevated in TIICs, implying that active cortisol may also be generated locally by immune cells (Fig. 7B, Supplementary Figure SF3B). Supporting this, GR-correlated genes within TIIC-rich regions were enriched for the glucocorticoid biosynthetic process (GO:0031946) (Supplementary Table ST1).

To further evaluate cell-cell communication, we performed integrated CellChat analysis across all four TNBC spatial transcriptomics samples. This revealed increased intercellular communication at tumour-stroma interface zones (Supplementary Figure SF3C). As previously observed that *TGFBR2* and *TGFB1* expression showed significant correlation with *NR3C1*, we focused on TGFβ signalling. We observed strong ligand–receptor interactions between tumour cells and TIICs, and between tumour cells and mixed immune compartments (Fig. 7C). Connective tissue and adipocytes showed minimal TGFβ-mediated communication.

Together, these results support that GR and TGFβ signalling are co-activated at the tumour-immune interface and may synergistically shape microenvironmental responses in TNBC. 

### Investigating the link between GR activation and TGFβ signalling

**Fig. 8:**
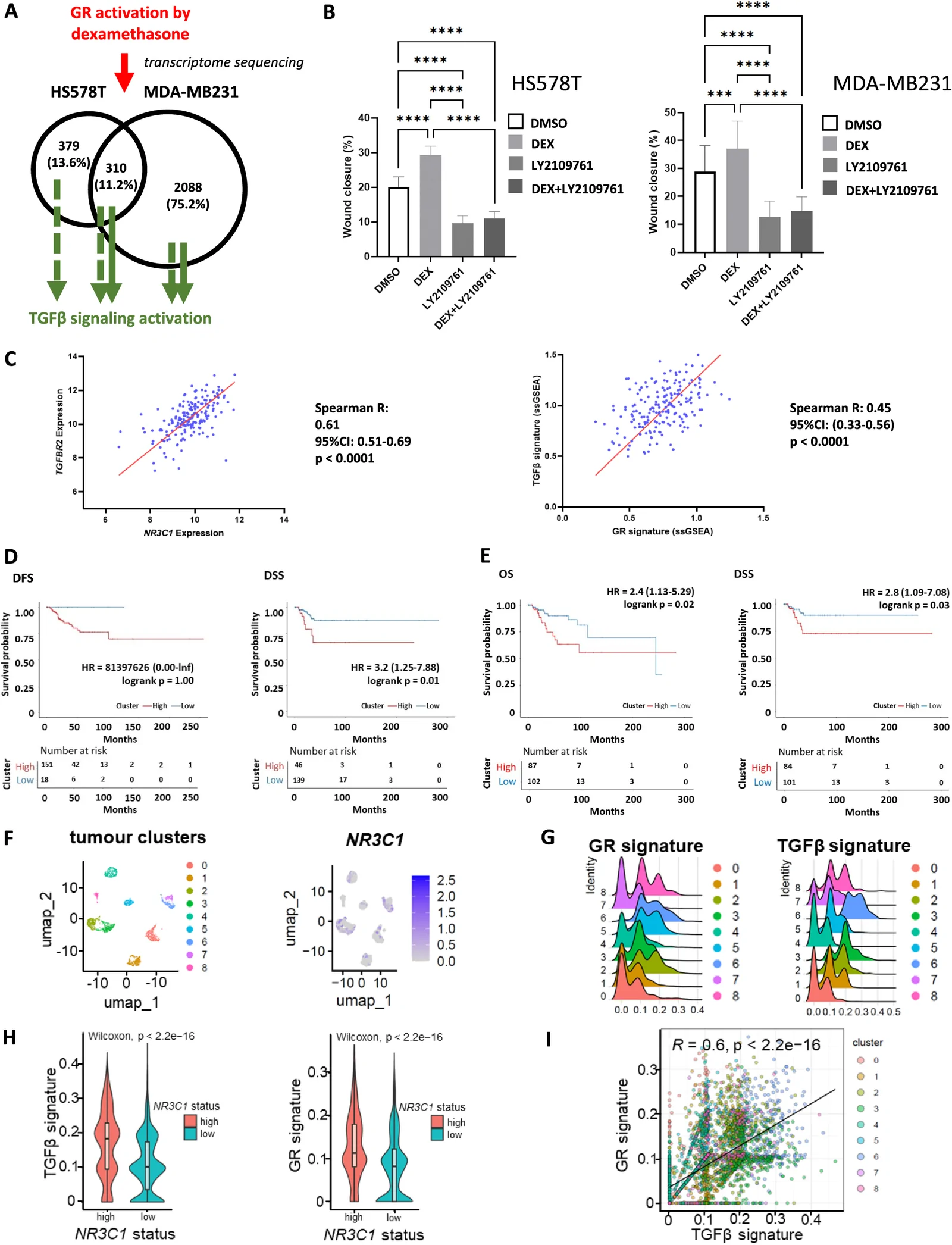
Functional characterisation of GR and TGFβ activation and their prognostic significance in TNBC. **A** Dexamethasone-induced activation of TGFβ signalling in HS578T and MDA-MB231 TNBC cell lines, as determined by transcriptome sequencing. The Venn diagram depicts differentially expressed genes between dexamethasone and DMSO conditions. Solid green arrows indicate significant enrichment of the TGFβ pathway; dashed arrows denote gene ontology biological processes involving TGFβ. **B** Wound-healing assays following 24-h treatment with dexamethasone (100 nM) with or without the TGFBR2 inhibitor LY2109761 (25 µM). Dexamethasone induced a mild but significant increase in cell migration, which was strongly suppressed by LY2109761. The inhibitor also abrogated the dexamethasone-induced promigratory effect when applied in combination. Columns represent mean ± SD of wound closure percentages. Statistical comparisons were made using ANOVA with Tukey’s post-hoc test (***p ≤ 0.001; ****p ≤ 0.0001). **C** Significant correlations between *NR3C1*, *TGFBR2*, and their gene signatures in the TCGA TNBC cohort (*n* = 189). Signatures were derived from transcriptomic profiling of dexamethasone-treated HS578T and MDA-MB231 cells. GR signature: 8 genes; TGFβ signature: 9 genes (detailed gene lists in the manuscript). R = Spearman correlation coefficient. **D** Survival analysis of GR-correlated genes identified by spatial transcriptomics in TNBC tumour cells using TCGA TNBC data (*n* = 189). The GR-correlated set included 107 genes commonly regulated across all four spatial specimens (Spearman *R* > 0.3, adjusted *p* < 0.05). DFS = disease-free survival; DSS = disease-specific survival; HR = hazard ratio. **E** Survival analysis of the dexamethasone-induced TGFβ signature (*JUN*,* THBS1*,* ITGB3*) in the TCGA TNBC cohort (*n* = 189). OS = overall survival; DSS = disease-specific survival; HR = hazard ratio. **F** Seurat-defined tumour clusters of TNBC samples showing differential NR3C1 expression. **G** Ridge plots depicting GR and TGFβ signature distributions across tumour clusters stratified by NR3C1 expression, analysed by ssGSEA. **H** Comparison of GR and TGFβ signature scores in *NR3C1*-high vs. *NR3C1*-low TNBC tumour cells. **I** Positive correlation between GR and TGFβ signatures at the single-cell level within TNBC tumour cells based on ssGSEA analysis

We next assessed changes in TGFβ signalling following GR activation in vitro. In our previous work, oestrogen receptor positive and negative cell lines were treated with the GR agonist dexamethasone to evaluate the transcriptional output of GR activation [24]. For the present analysis, we re-examined this dataset, focusing specifically on the triple-negative breast cancer cell lines HS578T and MDA-MB-231. Transcriptomic profiling after 24 h of 100 nM dexamethasone treatment revealed upregulation of TGFβ signalling in both cell lines, suggesting that TGFβ may act as a downstream effector of GR (Fig. 8A).

As we had previously shown that GR activation enhances TNBC cell motility, we next investigated the functional interplay between GR and TGFβ signalling using dexamethasone and the TGFBR2 inhibitor LY2109761. First, an optimisation experiment was conducted to determine the optimal concentration of LY2109761 to avoid cytotoxic effects (Supplementary Fig. 4A). Based on these results, in the further experiments, 25 µM LY2109761 was applied either individually or in combination with 100 nM dexamethasone. Subsequently, wound-healing assays demonstrated that dexamethasone induced a mild but significant increase in cell migration, whereas LY2109761 markedly inhibited migration and completely abolished the dexamethasone-induced effect when administered in combination (Fig. 8B, Supplementary Fig. 4B). Interestingly, the pS134-GR/TGFβ1-induced, ligand-independent 24-gene signature [13] in MDA-MB-231 cells was detectable under basal conditions and it did not show significant suppression by LY2109761 treatment (Supplementary Fig. 5). Similarly, LY2109761 did not suppress the signature in the presence of dexamethasone. Similar results were observed in Hs578T cells, although these data should be interpreted with caution because the signature was originally derived from MDA-MB-231 cells. 

### Clinical correlation and prognostic significance of GR and TGFβ co-activation in TNBC

To evaluate the clinical relevance of our findings, we examined the relationship between *NR3C1* and *TGFBR2* expression using the TCGA TNBC cohort. A strong positive correlation was observed between the two genes (Fig. 8C). To assess GR activation and its downstream effects in these samples, we derived GR and TGFβ pathway signatures based on our in vitro transcriptomic dataset, which included genes significantly altered following dexamethasone treatment and enriched for glucocorticoid receptor (GR) or TGFβ signalling pathways. The GR signalling signature comprised eight genes (*NR3C1*,* TSC22D3*,* SGK1*,* IL11*,* SPRY1*,* JUN*,* FGD4 and TNFAIP3*), while the TGFβ signalling signature consisted of nine genes (*SMAD3*,* JUN*,* FST*,* LIF*,* THBS1*,* CCND1*,* ITGA2*,* ITGB3 and FN1*). Single-sample GSEA (ssGSEA) revealed a strong positive correlation between these two signatures (Fig. 8C), suggesting that GR activation and TGFβ signalling are functionally linked in clinical TNBC specimens, consistent with our in vitro observations.

We next evaluated whether GR- and TGFβ-related transcriptional programs were associated with clinical outcome. GR-correlated genes were defined using spatial transcriptomics data derived exclusively from TNBC cells, including 107 genes consistently correlated across all four spatial specimens (Spearman’s *R* > 0.3, adjusted *p* < 0.05; Supplementary Table ST3, Fig. 8D). In parallel, we analysed genes from the dexamethasone-induced TGFβ signature identified in vitro, comprising *JUN*,* THBS1*, and *ITGB3* (Fig. 8E). Both GR-correlated and GR-induced TGFβ gene sets were significantly associated with poorer overall survival, indicating that GR-driven TGFβ activation may contribute to adverse patient outcomes.

To cross-validate the functional interplay between GR and TGFβ signalling, we reanalysed single-cell RNA-sequencing data from three independent TNBC studies [20–22]. Comparison of Seurat-defined tumour clusters revealed consistent GR and TGFβ signature patterns across clusters differing in *NR3C1* expression levels (Fig. 8F-G; Supplementary Fig. 6). Tumour cells with high *NR3C1* expression exhibited significantly elevated GR and TGFβ signature scores compared with *NR3C1* low-expressing cells (Fig. 8H; Supplementary Fig. 6). Furthermore, a positive correlation between GR and TGFβ signatures was also evident at the single-cell level within TNBC tumour cells in all three studies (Fig. 8I; Supplementary Fig. 6).

## Discussion

### GR is enriched in the TNBC tumour microenvironment

Conventional immunohistochemistry, single-cell RNA sequencing, and spatial transcriptomics consistently revealed heterogeneous GR expression across TNBC samples, with particularly strong GR positivity in stromal compartments. Tumour–normal comparisons demonstrated limited overlap in GR-associated transcriptional programs across TNBC cell types, indicating pronounced cell-type-specific GR activity within the tumour microenvironment (TME).

Cancer-associated fibroblasts and adipocytes are known to be reprogrammed by tumour cells to support tumour growth through metabolic and paracrine mechanisms [39, 40]. Accordingly, GR activity in tumour-adjacent fibroblasts was associated with cellular stress responses and apoptosis, while in adipocytes it was linked to lipid metabolism, development, differentiation, and mesenchymal stem cell migration. In tumour-infiltrating immune cells, GR regulated cytoskeletal organisation and immune-related functions, consistent with prior reports describing GR expression across multiple immune lineages [41].

In contrast, in normal breast tissue, GR activity was associated with epithelial morphogenesis, development, and proliferation, closely coordinated with oestrogen receptor (ESR1) signalling [39, 42, 43].

Within TNBC tumour cells, however, GR predominantly regulated cell proliferation, migration, immune signalling, and TGFβ-related pathways, indicating global reprogramming of GR signalling across all cellular compartments. Despite this heterogeneity, GR-correlated gene sets converged on shared processes including TGFβ signalling, epithelial–mesenchymal transition (EMT), cellular stress responses, and immune regulation.

Notably, GR activation – as indicated by nuclear localisation – was observed within tumours and adjacent normal breast tissue but was absent in distant normal breast or non-tumour specimens, consistent with tumour-associated field cancerization [44–46]. In line with emerging concepts of spatial tumour organisation [47], GR expression increased toward tumour edges, supporting a role for GR in invasion-related programs at the tumour–stroma interface.

### GR–TGFβ signalling crosstalk drives pro-migratory programs in TNBC

Single-cell and spatial transcriptomic analyses identified EMT and TGFβ signalling among the most consistently altered pathways shared between tumour and stromal compartments in TNBC. Spatial co-localisation of GR and TGFBR2 – most prominent in tumour cells and tumour-infiltrating immune cells – was confirmed by confocal microscopy and supported by spatial gene correlation analyses.

Functionally, dexamethasone treatment of TNBC cell lines induced robust activation of TGFβ signalling, consistent with prior reports [13]. We showed that pharmacological inhibition of TGFBR2 abolished dexamethasone-induced tumour cell migration, demonstrating that GR-driven motility is largely mediated through TGFβ signalling [13, 24]. GR promotes migration and aggressiveness through downstream effectors including *SGK1, MKP1, and ROR1*, which regulate adhesion, survival, MAPK signalling, and metastatic potential [8, 48, 49]. Interestingly, although TGFBR2 inhibition completely abolished dexamethasone-induced migration, it did not suppress the published pS134-GR/TGFβ1-induced, ligand-independent GR transcriptional signature. These findings suggest that TGFBR2 signalling is required for the dexamethasone-induced pro-migratory phenotype but is unlikely to exert its effects through global inhibition of this specific ligand-independent GR-associated transcriptional programme.

Beyond pathway induction, GR–TGFβ crosstalk is reinforced by p38 MAPK-mediated phosphorylation of GR at Ser134, enabling ligand-independent GR activation that drives migration, invasion, and chemoresistance [4, 13, 50]. This modification integrates ligand-dependent GR signalling and correlates with poor clinical outcomes [9, 51]. In TCGA TNBC samples, *NR3C1* and *TGFBR2* expression showed strong positive correlation, and GR- and TGFβ-related transcriptional signatures were tightly linked and associated with reduced overall survival. These findings were independently validated at single-cell resolution across multiple TNBC cohorts, establishing GR–TGFβ co-activation as a clinically relevant, tumour cell–intrinsic program.

### GR-TGFβ signalling in tumour-microenvironment cell-cell interactions

Local glucocorticoid activity depends on cortisol release from corticosteroid-binding globulin (CBG) and intracellular activation by HSD11B1 [37]. Neutrophil elastase (*ELANE*) is the principal enzyme mediating CBG cleavage. In normal breast tissue, *ELANE* and *HSD11B1* expression was confined largely to stromal fibrocytes, whereas TNBC exhibited enrichment of *ELANE* in tumour-infiltrating immune cells. Concurrently, tumour cells displayed high *HSD11B2* and low *HSD11B1* expression, consistent with ligand-independent GR activation. Elevated *HSD11B1* in immune cells further suggests local cortisol generation within the TNBC microenvironment.

These findings highlight highly compartment-specific regulation of glucocorticoid signalling in TNBC, whereby tumour cell–intrinsic GR activation can be sustained independently of systemic cortisol, while immune compartments contribute to local glucocorticoid bioavailability. Given that immune cells express high GR levels and exhibit altered immune phenotypes in GR-high tumours [4, 11, 52], GR signalling likely influences both tumour progression and immune contexture.

Consistent with this, spatial cell–cell communication analysis revealed enhanced signalling at tumour–stroma interface regions, with TGFβ emerging as a dominant mediator of tumour–immune interactions. Given the established roles of TGFβ in immune suppression, angiogenesis, EMT, and tumour progression [4, 53], coordinated GR–TGFβ signalling may critically shape microenvironmental remodelling, tumour cell migration, and therapeutic response.

### Conclusion and integrated perspective

Spatial transcriptomics has established that TNBC is organised into distinct spatial ecosystems that govern intratumour heterogeneity, progression, and treatment response [27, 54–56]. Building on this framework, our data identify spatially resolved GR activation and its functional coupling with TGFβ signalling as an additional regulatory layer. We demonstrate that treatment-naïve TNBC exhibits tumour cell–intrinsic, ligand-independent GR activation, whereas GR signalling in stromal and immune compartments is predominantly ligand-mediated. Given the widespread clinical use of synthetic glucocorticoids, these findings underscore the need for careful evaluation of glucocorticoid administration and highlight the GR–TGFβ axis as a potential therapeutic vulnerability in TNBC. 

## Supplementary Information

Supplementary Material 1. Supplementary Figure 1: GR expression in different breast cancer cell types revealed by single cell sequencing. (A) Cell-type annotation of single-cell data from Pal et al. including TNBC and normal samples. (B) Tumour-versus-normal annotation of Seurat clusters. (C) GR-correlated genes display distinct expression patterns and numbers of differentially expressed genes between tumour and normal tissues across multiple cell types. Statisctics were done by Wilcoxon test, *: *p*<0.05, **: 0<0.01. Supplementary Figure 2: Functional differences in GR-correlated genes across tumour and stromal compartments. (A) Gene set enrichment analysis of biological processes in spatial transcriptomics data reveals cell-type-specific roles of GR activation in TNBC tumour and stromal cells. (B) Tree plot comparing the biological functions of GR-correlated genes in TNBC tumour versus normal breast epithelial regions. Supplementary Figure 3: Local cortisol availability, activation and tumour–stroma signalling. (A) For the analysis of normal breast tissue, single-cell sequencing data of 47662 individual cells of normal breast tissue from the Human Protein Atlas were used. Cluster annotation indicated by the different colours was used as given by Human Protein Atlas. (B) *ELANE*, *HSD11B1* and *HSD11B2* gene expression in TNBC tissues in our spatial transcriptomics experiment in all four samples visualized on violin plots. Statistical comparisons were done by Kruskal- Wallis analysis of variance with Dunn’s posthoc test, where *: *p*<0.05. (C) CellChat analysis demonstrated increased overall intercellular signalling at tumour–stroma interface regions, where multiple cell types were in close proximity and direct contact, suggesting an active communication network between tumour and stromal components. Supplementary Figure 4: Optimisation of TGFBR2 inhibitor concentration and representative images of cell migration assays. (A) Optimisation of LY2109761 concentration was performed using a 10-point dilution series (10^-8^–10^2^ μM) to determine the highest non-cytotoxic dose via sulforhodamine B (SRB) cell viability assays. A concentration of 25 μM LY2109761 was selected for migration assays, as cells maintained high viability at this dose. (B) Representative images from collective migration (wound-healing) assays of HS578T and MDA-MB231 TNBC cells treated with the GR agonist dexamethasone (100 nM) and/or the TGFBR2 inhibitor LY2109761 (25 μM). Images depict wound areas immediately after scratching (0 h) and after 6 h of treatment. See Methods and Results sections for details. Supplementary Figure 5: Enrichment of 24-gene pS134-GR/TGFβ1-induced signature proposed as a surrogate for ligand-independent GR signalling after dexamethasone and TGFBR2 inhibitor treatment. Significant changes were observed only in the DMSO vs. DEX comparison in the MDA-MB231 cell line using the Student's t-Test. Supplementary Figure 6: Correlation of GR and TGFβ signatures in TNBC revealed by single-cell RNA sequencing. Single-cell RNA sequencing datasets from three independent TNBC studies – Pal et al. (A), Wu et al. (B), and Bassez et al. (C) – were re-analysed. Seurat clustering identified tumour cell populations differing in *NR3C1* expression. Across all datasets, GR and TGFβ signatures displayed similar distribution patterns in ridge plots analysed by ssGSEA, differing according to *NR3C1* expression level. Comparisons between *NR3C1*-high and NR3C1-low tumour cells revealed significantly higher GR and TGFβ signature scores in the *NR3C1*-high groups. A significant positive correlation between GR and TGFβ signatures at the single-cell level was consistently observed across all three studies.

Supplementary Material 2. Supplementary Table ST1: Gene set enrichment analysis of the top 250 genes showing the strongest correlation with glucocorticoid receptor (GR) expression across spatially annotated spots representing distinct cellular compartments. *Abbreviations*: T_TNBC, spots containing cells annotated as triple-negative breast cancer tumour cells; TME_Adipocyte, spots containing adipocytes adjacent to tumour cells; TME_CT, spots containing connective tissue; TME_TIL, spots containing tumour-infiltrating lymphocytes.

Supplementary Material 3. Supplementary Table ST2: Functional enrichment analyses from spatial transcriptomics. ST2A. Significantly enriched biological functions and pathways among differentially expressed genes between normal breast epithelium and triple-negative breast cancer in the spatial transcriptomics analysis. Enrichment related to glucocorticoid receptor signalling and TGFβ signalling is highlighted in yellow and pink, respectively. ST2B. Significant functional enrichment of GR-correlated genes uniquely identified in normal breast epithelium or triple-negative breast cancer in the spatial transcriptomics analysis. Immune-associated functions and TGFβ signalling pathways are highlighted. ST2C. Significant functional enrichment of GR-transactivated genes shared across all four TNBC samples in the spatial transcriptomics analysis. Glucocorticoid-related terms and pathways are highlighted in yellow, and TGFβ-related terms in pink.

Supplementary Material 4. Supplementary Table ST3: Association of tumour cell-specific GR gene signature with patient outcomes. Only genes (*n* = 107) commonly regulated across all four spatial specimens were included (Spearman R > 0.3, adjusted *p* < 0.05). *Abbreviations: OS, overall survival; DFS, disease-free survival; PFS, progression-free survival; DSS, disease-specific survival; HR, hazard ratio*.

## Data Availability

All data generated or analysed during this study are included in this published article. Raw transcriptomic data will be made available upon manuscript acceptance through a publicly available repository.
