# Supplementary material for: Spatially resolved GR-TGFβ co-activation underlies cell-type-specific signalling in triple-negative breast cancer
Source: Cell Commun Signal. 2026 Jul 28;24:592. doi: 10.1186/s12964-026-03104-7 (PMC13729666; doi:10.1186/s12964-026-03104-7)
Supplement: Supplementary file 1 [file 12964_2026_3104_MOESM1_ESM.docx]

**Supplementary Figure 1.** **GR expression in different breast cancer cell types revealed by single cell sequencing**

(**A**) Cell-type annotation of single-cell data from Pal et al. including TNBC and normal samples

(**B**) Tumour-versus-normal annotation of Seurat clusters

(**C**) GR-correlated genes display distinct expression patterns and numbers of differentially expressed genes between tumour and normal tissues across multiple cell types. Statisctics were done by Wilcoxon test, *: p<0.05, **: 0<0.01

**Supplementary Figure 2. Functional differences in GR-correlated genes across tumour and stromal compartments**

(**A**) Gene set enrichment analysis of biological processes in spatial transcriptomics data reveals cell-type-specific roles of GR activation in TNBC tumour and stromal cells.

(**B**) Tree plot comparing the biological functions of GR-correlated genes in TNBC tumour versus normal breast epithelial regions.

**Supplementary Figure 3.** **Local cortisol availability, activation and tumour–stroma signalling**

(**A**) For the analysis of normal breast tissue, single-cell sequencing data of 47662 individual cells of normal breast tissue from the Human Protein Atlas were used. Cluster annotation indicated by the different colours was used as given by Human Protein Atlas.

(**B**) *ELANE, HSD11B1* and *HSD11B2* gene expression in TNBC tissues in our spatial transcriptomics experiment in all four samples visualized on violin plots. Statistical comparisons were done by Kruskal- Wallis analysis of variance with Dunn’s posthoc test, where *: p<0.05.

(**C**) CellChat analysis demonstrated increased overall intercellular signalling at tumour–stroma interface regions, where multiple cell types were in close proximity and direct contact, suggesting an active communication network between tumour and stromal components.

**Supplementary Figure 4.** **Optimisation of TGFBR2 inhibitor concentration and representative images of cell migration assays**

(**A**) Optimisation of LY2109761 concentration was performed using a 10-point dilution series (10⁻⁸–10² μM) to determine the highest non-cytotoxic dose via sulforhodamine B (SRB) cell viability assays. A concentration of 25 μM LY2109761 was selected for migration assays, as cells maintained high viability at this dose.

(**B**) Representative images from collective migration (wound-healing) assays of HS578T and MDA-MB231 TNBC cells treated with the GR agonist dexamethasone (100 nM) and/or the TGFBR2 inhibitor LY2109761 (25 μM). Images depict wound areas immediately after scratching (0 h) and after 6 h of treatment. See Methods and Results sections for details.

**Supplementary Figure 5. Enrichment of 24-gene pS134-GR/TGFβ1-induced signature proposed as a surrogate for ligand-independent GR signalling after dexamethasone and TGFBR2 inhibitor treatment. Significant changes were observed only in the DMSO vs. DEX comparison in the MDA-MB231 cell line using the Student's t-Test.**

**Supplementary Figure 6. Correlation of GR and TGFβ signatures in TNBC revealed by single-cell RNA sequencing**

Single-cell RNA sequencing datasets from three independent TNBC studies – Pal et al. (**A**), Wu et al. (**B**), and Bassez et al. (**C**) – were re-analysed.

Seurat clustering identified tumour cell populations differing in *NR3C1* expression. Across all datasets, GR and TGFβ signatures displayed similar distribution patterns in ridge plots analysed by ssGSEA, differing according to *NR3C1* expression level.

Comparisons between *NR3C1*-high and *NR3C1*-low tumour cells revealed significantly higher GR and TGFβ signature scores in the *NR3C1*-high groups. A significant positive correlation between GR and TGFβ signatures at the single-cell level was consistently observed across all three studies.

**Supplementary Table ST1. Gene set enrichment analysis of the top 250 genes showing the strongest correlation with glucocorticoid receptor (GR) expression across spatially annotated spots representing distinct cellular compartments**

*Abbreviations:* T_TNBC, spots containing cells annotated as triple-negative breast cancer tumour cells; TME_Adipocyte, spots containing adipocytes adjacent to tumour cells; TME_CT, spots containing connective tissue; TME_TIL, spots containing tumour-infiltrating lymphocytes.

**Supplementary Table ST2. Functional enrichment analyses from spatial transcriptomics**

**ST2A.** Significantly enriched biological functions and pathways among differentially expressed genes between normal breast epithelium and triple-negative breast cancer in the spatial transcriptomics analysis. Enrichment related to glucocorticoid receptor signalling and TGFβ signalling is highlighted in yellow and pink, respectively.

**ST2B.** Significant functional enrichment of GR-correlated genes uniquely identified in normal breast epithelium or triple-negative breast cancer in the spatial transcriptomics analysis. Immune-associated functions and TGFβ signalling pathways are highlighted.

**ST2C.** Significant functional enrichment of GR-transactivated genes shared across all four TNBC samples in the spatial transcriptomics analysis. Glucocorticoid-related terms and pathways are highlighted in yellow, and TGFβ-related terms in pink.

**Supplementary Table ST3.** Association of tumour cell-specific GR gene signature with patient outcomes.

Only genes (n = 107) commonly regulated across all four spatial specimens were included (Spearman R > 0.3, adjusted p < 0.05).

*Abbreviations: OS, overall survival; DFS, disease-free survival; PFS, progression-free survival; DSS, disease-specific survival; HR, hazard ratio*
