# Supplementary material for: Spatially resolved GR-TGFβ co-activation underlies cell-type-specific signalling in triple-negative breast cancer
Source: Cell Commun Signal. 2026 Jul 28;24:592. doi: 10.1186/s12964-026-03104-7 (PMC13729666; doi:10.1186/s12964-026-03104-7)
Supplement: Supplementary file 2 [file 12964_2026_3104_MOESM4_ESM.docx]

**Supplementary Table ST3.** Association of tumour cell-specific GR gene signature with patient outcomes.

Only genes (n = 107) commonly regulated across all four spatial specimens were included (Spearman R > 0.3, adjusted p < 0.05).

*Abbreviations: OS, overall survival; DFS, disease-free survival; PFS, progression-free survival; DSS, disease-specific survival; HR, hazard ratio*

| OS | | DFS | | PFS | | DSS | |
| --- | --- | --- | --- | --- | --- | --- | --- |
| HR (95%CI) | adj.p value | HR (95%CI) | adj.p value | HR (95%CI) | adj.p value | HR (95%CI) | adj.p value |
| 2.2 (0.96-5.17) | *0.06* | 881397626 (0.00-lnf) | 0.00 | 2.0 (0.89-4.27) | 0.09 | 3.2 (1.26-7.88) | 0.01 |
